# Minimally invasive intrathecal spinal cord imaging with optical coherence tomography

**DOI:** 10.1117/1.JBO.26.5.056002

**Published:** 2021-05-13

**Authors:** Christopher R. Pasarikovski, Jerry C. Ku, Joel Ramjist, Yuta Dobashi, Stefano M. Priola, Leodante da Costa, Ashish Kumar, Victor X. D. Yang

**Affiliations:** a University of Toronto, Division of Neurosurgery, Department of Surgery, Toronto, Ontario, Canada; b University of Toronto, Sunnybrook Hospital, Division of Neurosurgery, Toronto, Ontario, Canada; c Health Sciences North, Division of Neurosurgery, Department of Surgery, Sudbury, Ontario, Canada; d University of Toronto, Sunnybrook Research Institute, Hurvitz Brain Sciences Research Program, Toronto, Ontario, Canada; e University of Toronto, Sunnybrook Health Sciences Centre, Toronto, Ontario, Canada

**Keywords:** optical coherence tomography, imaging, spinal canal, spinal cord

## Abstract

**Significance:** Imaging of the spinal cord is challenging due to the surrounding bony anatomy, physiologic motion, and the small diameter of the spinal cord. This precludes the use of non-invasive imaging techniques in assessing structural changes related to trauma and evaluating residual function.

**Aim:** The purpose of our research was to apply endovascular technology and techniques and construct a preclinical animal model of intrathecal spinal cord imaging using optical coherence tomography (OCT).

**Approach:** Five animals (2 Yorkshire Swine and 3 New Zealand Rabbits) were utilized. Intrathecal access was gained using a 16-guage Tuohy, and an OCT catheter was advanced under roadmap technique into the cervical canal. The OCT catheter has a motorized pullback, and a total length of 54 mm of the spinal canal is imaged.

**Results:** Image acquisition was successful for all animals. There were no instances of difficult catheter navigation, enabling OCT imaging rostrally to C2. The thecal sac provided excellent thoroughfare for the OCT catheter. The clear cerebrospinal fluid also provided an excellent medium for image acquisition, with no detectable artifact from the contents of the cerebrospinal fluid. The anatomical space of the spinal canal could be readily appreciated including: dural lining of the thecal sac, epidural veins, pial lining of the spinal cord, arachnoid bands, dentate ligaments, and nerve rootlets/roots.

**Conclusion:** Minimally invasive intrathecal imaging using endovascular OCT was feasible in this preclinical animal study. The repurposing of an endovascular device for spinal imaging comes with limitations, and a spine-specific device is necessary.

## 1 Introduction

Diagnostic imaging of the spinal canal has evolved considerably over the past two decades.^1^ Non-invasive imaging modalities are the radiological tools of choice for the majority of spinal pathologies including: trauma, tumors, infections, and inflammatory disease.^2^ In spinal trauma, computed tomography (CT) is very sensitive in the detection of fractures or dislocations, and magnetic resonance (MR) imaging can reliably identify cord compression, transections, edema, and hemorrhages.^3^ Although significant progress has been made at assessing structural changes related to trauma and evaluating residual function, non-invasive spinal cord imaging modalities continue to be hindered by susceptibility differences, motion secondary to cardiac function, and the small cross-sectional area of the spinal cord leading to limited clinical utility.^4^^–^^7^

In 1991, a technology called optical coherence tomography (OCT) was developed by Huang et al.^8^ who first demonstrated the technique and its application on the human retina and coronary artery *in vitro.* The cross-sectional images generated using OCT utilize backscattered light from the tissue structure. The underlying principal is that various biological tissues in the body have varying optical indices, and therefore, different tissue layers will reflect the light at different amplitudes. A spatial resolution of 10 to 15  μm is achievable. The depth of penetration is ∼3  mm.

In comparison, intravascular ultrasound has a spatial resolution of 100  μm (∼10 times less resolution), and 3-Tesla MR imaging has a voxel size of 2.0×0.4×0.4  mm. With near histological resolution, OCT has been described as an optical biopsy modality.^9^ The United States Food and Drug Administration approved the use of endovascular OCT for the diagnosis and treatment of cardiovascular disease in 2010. The catheter can be navigated to the site of interest in a monorail fashion over a 0.014-arc sec microwire, using the vasculature as thoroughfare.

The spinal cord is located in the spinal canal within the thecal sac, which is filled with clear cerebrospinal fluid. The cerebrospinal fluid could conceivably be used as a media to navigate an OCT catheter to various anatomical locations within the spinal column. The thecal sac would act as a highway, analogous to the vessel lumen in cardiac imaging. The objective of advancing spinal cord imaging is rooted in improving clinician’s ability to accurately diagnose, treat injuries, and diseases and predict clinical outcomes. The aim of this research was to construct a preclinical animal model of intrathecal spinal cord imaging using OCT. The goal is to determine if OCT catheter navigation through the thecal sac is feasible, and if cross-sectional images of the spinal cord can be generated through the cerebrospinal fluid.

## 2 Methods

All experiments were conducted according to the policies and standards established by the authors’ institutional animal research ethics board. Five animals total, two Yorkshire Swine weighing 40 to 45 kg and three New Zealand White Rabbits weighting 5 kg, were utilized for spinal OCT imaging. The rationale for using two different species was testing if the catheter could be navigated in animals with larger and smaller dimension spinal canals. There was no prescreening imaging for any animal. All procedures were carried out under general anesthetic with continuous hemodynamic monitoring. Imaging data in this study are available from the corresponding author upon written request.

### 2.1 Subarachnoid Access

**Fig. 1:**
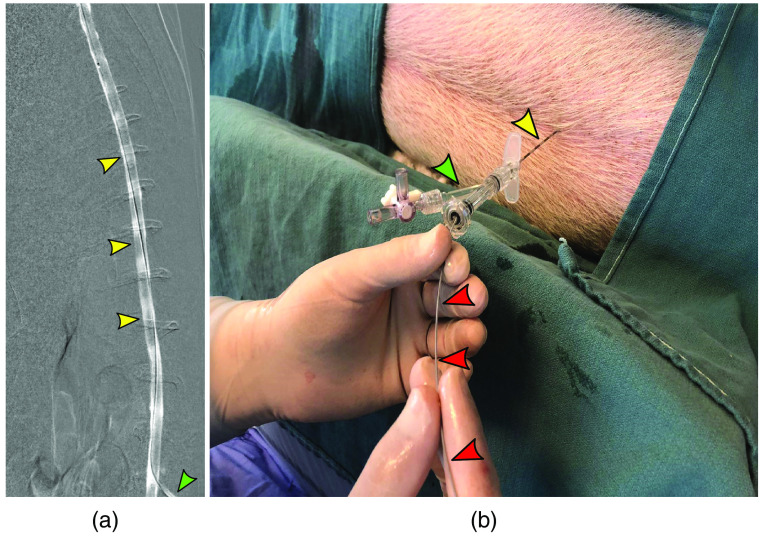
Subarachnoid access: (a) a 16-guage Tuohy needle (green arrows) was advanced into the lumbar cistern and contrast was injected to confirm subarachnoid positioning (yellow arrows). (b) With the Tuohy needle in position (yellow arrow) with an RHV (green arrow), the OCT catheter (red arrows) is fed gently through the RHV and needle into the lumbar cistern. To image the levels above, the Tuohy bevel is directed upwards to guide the OCT catheter under fluoroscopic guidance. OCT, optical coherence tomography.

A dedicated animal interventional radiology suite equipped with a single-plane C-Arm (Philips, Andover, MA) was used for all procedures. Under fluoroscopic guidance, the midline and L2/L3 level was landmarked, and a 16-guage Tuohy needle was advanced into the spinal canal. Once in position, the inner cannula was removed to confirm cerebrospinal fluid egression. Next, Omnipaque 300 (General Electric, Boston, Massachusetts) was injected via the Tuohy to further confirm the intrathecal positioning of the needle and provide a roadmap for OCT catheter navigation [Fig. 1(a)].

### 2.2 Optical Coherence Tomography Imaging

**Fig. 2:**
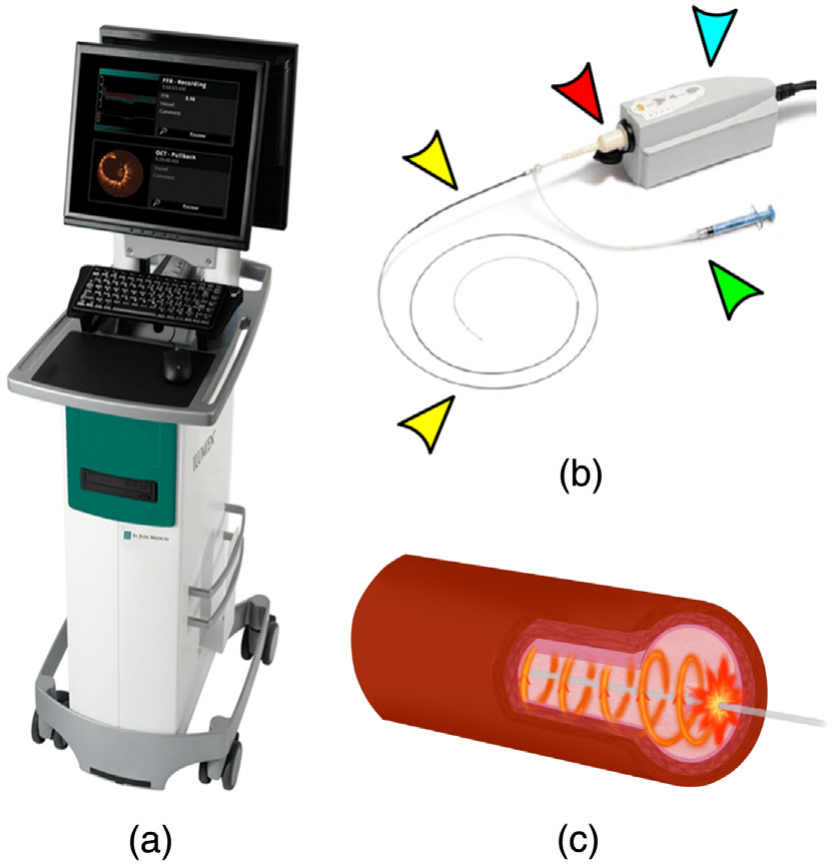
The ILUMIEN OPTIS system and Dragonfly OCT catheter. (a) The acquired OCT images are viewed directly on the mobile unit shown. (b) The OCT catheter (yellow arrows) has a large port (red arrow) allowing connection to a docking station (blue arrow) which is connected to the mobile unit. A 5-ml syringe (green arrow) is connected to the catheter to clear the lumen during use. (c) OCT cross-sectional images are acquired at 100 frames per second, with a total of 540 cross-sectional images generated per pullback. OCT, optical coherence tomography.

**Fig. 3:**
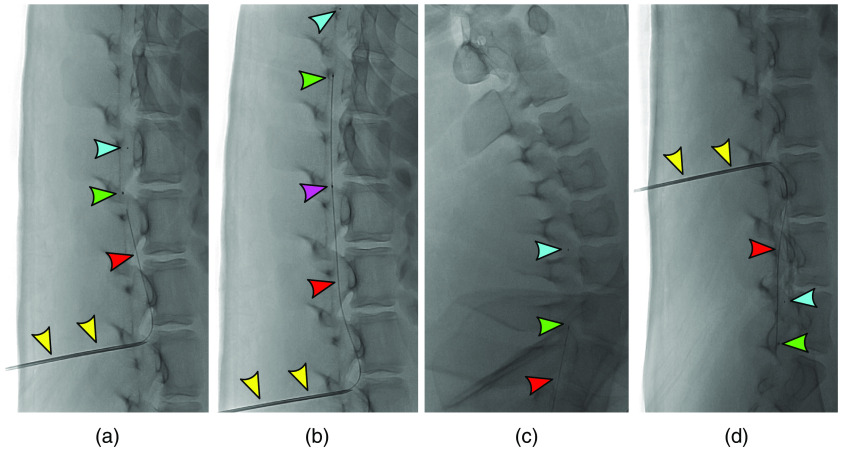
Fluoroscopic OCT catheter guidance. (a), (b) The Tuohy needle (yellow arrow) is shown at the L2/3 level. The OCT catheter has one distal marker (blue arrow), one lens marker (green arrow), and one proximal marker (purple arrow). The anatomic region that will be scanned is between the green and purple arrows and is 54 mm. The optical fiber is visible throughout (red arrow). (c) The catheter was advanced into the cervical spine without difficulty. (d) With the bevel directed inferiorly, the catheter was also navigated toward the sacrum without difficulty. OCT, optical coherence tomography.

The Dragonfly™ OCT catheter (Abbott Vascular, Chicago, Illinois) was used for all procedures (Fig. 2). This device generates cross-sectional images with spatial resolution of 10  μm. The depth of tissue penetration is ∼3  mm. Before insertion, the OCT catheter is connected to a saline flush to ensure constant clearing of debris. The catheter was gently fed through the rotary hemostatic valve (RHV) and Tuohy into the thecal sac [Figs. 1(b) and 2(b)]. The distal catheter is 2.7 French (0.9-mm diameter). To position the catheter in the region of interest, radiopaque catheter markers are used. There is one radiopaque marker located near the tip of the catheter and one marker at the lens spaced 23 mm apart (Fig. 3). The lens marker is located 50 mm distal to the proximal marker. Once the catheter is in position, it is connected to a docking station, which is connected to a mobile imaging processing station (Fig. 2).

Normally, the OCT catheter can be navigated to the site of interest in either a monorail fashion over a 0.014-arc sec microwire or coaxially through a larger distal access catheter when in the vasculature. Given the gentle curvature of the spinal canal without any sharp turns, the catheter could be navigated without a wire or distal access catheter. First, the catheter was advanced superiorly into the cervical spine where imaging was performed, and subsequently directed inferiorly toward the sacrum for imaging, by changing the orientation of the Tuohy bevel tip (Fig. 3).

Once in position the OCT catheter is connected to the docking station, image acquisition is enabled and motorized pullback is initiated. The catheter has a motorized pullback, and a total length of 54 mm is scanned with each pullback. OCT imaging frequency is 100 frames per second, with a total of 540 cross-sectional images generated per pullback. All cross-sectional OCT images were analyzed. Given the cerebrospinal fluid is clear, no clearing of the thecal space was needed. This is contrary to intravascular imaging, where an injection of contrast or saline is needed to clear the blood in the vessel lumen during image acquisition.

## 3 Results

A total of five animals (two swine and three rabbits) underwent intrathecal spinal cord imaging using the endovascular OCT catheter. Image acquisition was technically successful for all animals. Lumbar puncture via Tuohy needle was achieved, and the OCT catheter was passed through the needle and navigated throughout the spinal canal successfully in all cases. There were no instances of difficult catheter navigation cranially, enabling OCT imaging as rostral as C2. Caudally, the catheter could be navigated to the superior endplate of S1, at which point it would loop rostrally. The thecal sac provided excellent thoroughfare for the OCT catheter. The clear cerebrospinal fluid also provided an excellent medium for image acquisition, with no detectable artifact from the contents of the cerebrospinal fluid. There were no obvious complications such perforation of the thecal sac or obvious traumatic damage to the spinal cord in any animal.

**Fig. 4:**
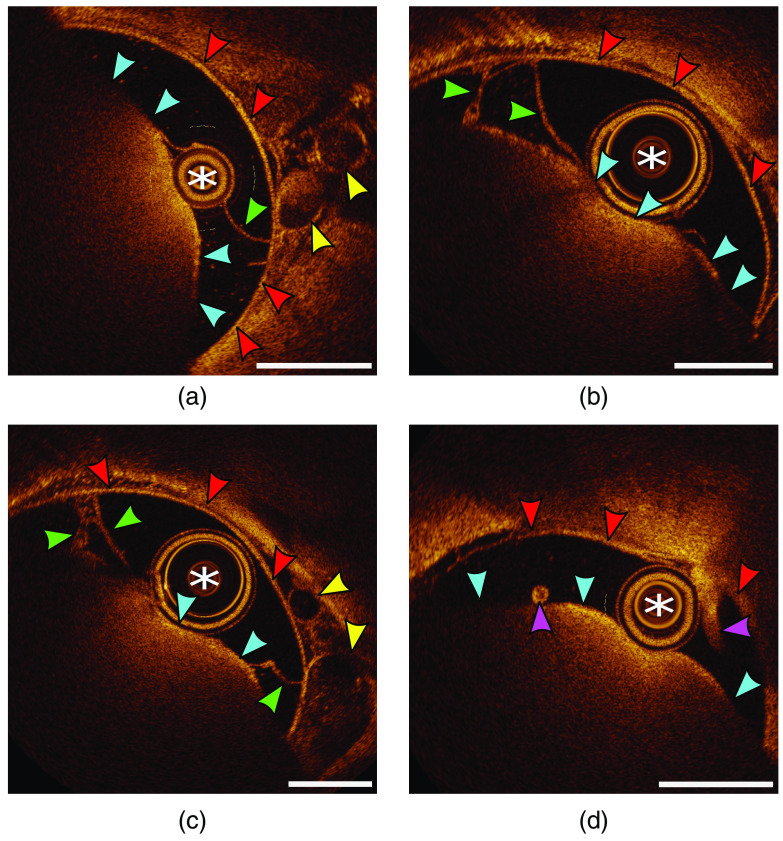
Intrathecal cervical spine OCT imaging. (a), (b) The OCT lens (white asterisk) located within the subarachnoid space, with cross-sectional imaging displaying the pial lining of the spinal cord (blue arrows), arachnoid bands and a lateral dentate ligament (green arrows), dura (red arrows), and epidural veins (yellow arrows). (c) Larger epidural veins (yellow arrows) are appreciated with a thick lateral dentate ligament (green arrow). (d) Two spinal nerves (purple arrows) within the subarachnoid space. OCT, optical coherence tomography. White bars=1  mm.

**Fig. 5:**
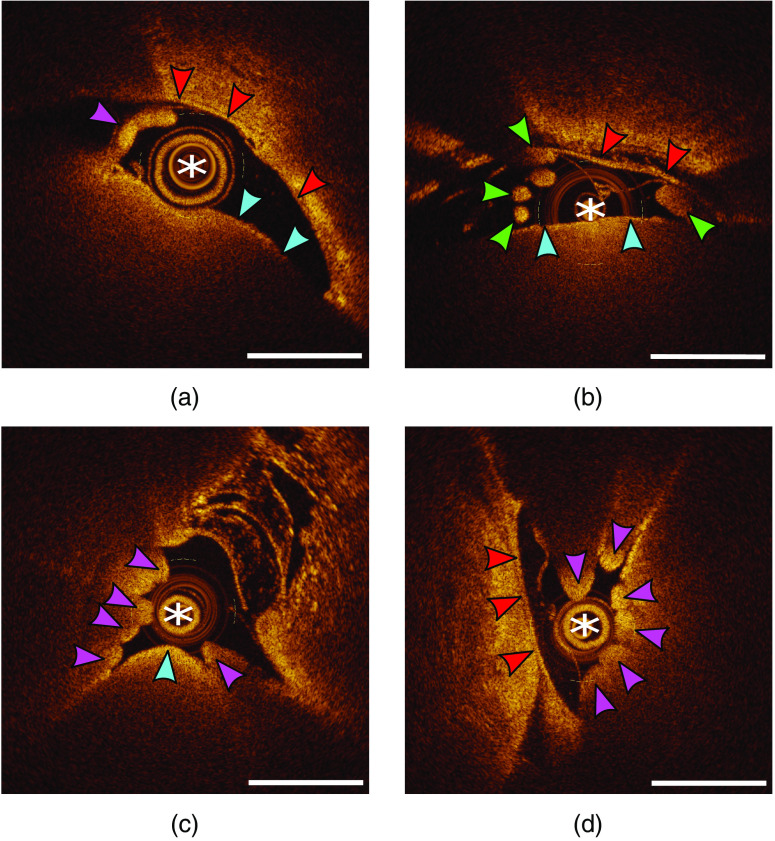
Intrathecal thoracic/lumbar spine OCT imaging. (a) The OCT lens (white asterisk) located within the subarachnoid space, with cross-sectional imaging displaying the pial lining of the low-thoracic spinal cord (blue arrows), dura (red arrows), and a nerve (purple arrow) coursing off the cord and through the thecal sac. (b) Multiple rootlets (green arrows) on the left and a nerve root on the right (green arrow). (c) The pial lining of the conus medullaris along with multiple rootlets of the cauda equina (purple arrows). (d) The dural lining (red arrows) of the lumbar cistern and cauda equina (purple arrows). OCT, optical coherence tomography. White bars=1  mm.

The anatomical space of the thecal sac could be readily appreciated with the OCT lens surrounded by cerebrospinal fluid. The dural lining of the thecal sac was visualized, along with epidural veins (Figs. 4 and 5). Various caliber veins could be appreciated. The pial lining of the spinal cord could be appreciated (Figs. 4 and 5). Anatomic spinal cord structures (dorsal/ventral horns and white matter tracks) beyond the pial lining could not be visualized. Arachnoid bands and dentate ligaments were visualized through the spinal column (Figs. 4 and 5). In the cervical spine, nerve rootlets were visualized before merging to form the nerve root (Fig. 5). Caudally, the conus medullaris was observed, with the cauda equina readily pictured (Fig. 5).

## 4 Discussion

The authors describe to the best of their knowledge the first intrathecal imaging of the spinal canal using OCT. This imaging acquisition technique is minimally invasive and allows for the highest spatial resolution currently available. The imaging was performed in a preclinical setting in swine and rabbits *in vivo*. An intravascular OCT imaging catheter designed and approved for the diagnosis and treatment of cardiac patients was repurposed and navigated throughout the thecal sac for spinal imaging. We found the navigation of the OCT catheter and the acquisition of cross-sectional images to be feasible in all five animals.

The advancement of spinal cord imaging with respect to the characterization of residual function and structure after spinal cord injury is imperative moving forward.^10^^–^^12^ Researchers have also tried to apply advanced MR techniques such as diffusion tensor imaging (DTI) to detect microstructural changes in spinal tumors and provide a preoperative evaluation of tumor respectability.^13^ In 2013, leaders from across the globe gathered for the first ever spinal cord imaging meeting with the aim of discussing the state-of-the-art for spinal cord imaging.^4^ Stroman et al.^4^ reported that the greatest challenge in spinal cord imaging was the anatomic location and surrounding environment of the spinal cord. The adjacent bony structures, physiologic motion of the cord, and the small area of the spinal cord all lead to difficulties for non-invasive imaging modalities. The committee proposed that researchers improve CT detector technology and MR radio-coil design and spectroscopy methods to reduce sensitivity to magnetic field inhomogeneity.^4^

In this feasibility study, the authors sought to determine if endovascular devices and techniques could be applied in spinal cord imaging. If imaging could be performed from within the thecal sac, one could eliminate artifacts from the surrounding bony structures. Furthermore, an imaging modality such as OCT with a spatial resolution of 10  μm could overcome the spatial limitations of CT and MR. These ideas directed the authors to attempt intrathecal catheterization and OCT catheter imaging. Cadott et al. (2012)^14^ first described the use of depth-resolved speckle variance OCT of the spinal cord of rats and mice. They utilized an open surgical approach performing a laminectomy and directly exposing the spinal cord for imaging. It would be challenging to justify such an invasive approach for diagnostic purposes, given the morbidity (hemorrhage, infection, soft tissue damage, and neural element injury) associated with this degree of surgical exposure. The proposed minimally invasive approach obviates the need for open surgery.

Although minimally invasive, as in this case with a 16-gauge Tuohy needle, thecal sac access and threading of an intrathecal catheter does not come without risk. Ahmadieh et al. (2020)^15^ conducted a trial of lumbar drainage for patients with normal pressure hydrocephalous. A total of 254 patients were included and complications for lumbar drainage comprised: 5 (2%) cerebrospinal fluid leaks, 3 (1.1%) meningitis, 1 (0.4%) epidural abscess, and 1 (0.4%) transient lower extremity paresthesia.^15^ We hypothesize that infectious complications in a 20-min imaging procedure could be lower compared to studies leaving a lumbar drain catheter *in situ* for four continuous days. However, lumbar drains are not typically advanced into the cervical spinal canal, generally only extending a few levels above the entry point in the lumbar spine at L3/L4. Conversely, intrathecal baclofen pump placement is a common surgical procedure for spasticity. The catheters are typically placed at the T10 level for diplegia, C5 for spastic tetraplegia, and C2 for generalized secondary dystonia.^16^ These catheters are introduced in a similar fashion via a Tuohy needle in the lumbar spine and advanced into the cervical canal.^16^ The diameter of these catheters is 4.2 French (1.2 mm), which is larger than the current OCT catheter at 2.7 French (0.9 mm). Coffey et al.^17^ reported no new neurological complications for 75 patients who underwent intrathecal catheter placement for intractable spasticity. Rekand and Gronning^18^ reported no neurological complications in 14 patients with multiple sclerosis undergoing intrathecal catheter placement with a mean follow-up of 62 months.

The authors did not assess neurological function postprocedure in this feasibility study, and therefore, cannot comment on the clinical safety of navigating a 0.9-mm catheter into the thoracic and cervical spine. Preclinical neurological outcome studies are required before any human investigations can be performed using OCT catheters. However, the authors hypothesize that given no neurological complications have been reported during the implantation and advancement of a 1.2-mm catheter from the lumbar to cervical spine during intrathecal baclofen pump surgery, navigation of smaller soft catheters may also be safe. Furthermore, this study was performed on anatomically normal spinal cords in swine and rabbits, and therefore imaging in pathologic states such as spinal trauma or infection may be more challenging.

Using OCT, the authors observed excellent visualization of the dura, subarachnoid space, epidural vessels, dentate ligaments, and nerve roots and rootlets. This could theoretically allow for high resolution, minimally invasive imaging for pathologies such as intradural extramedullary tumors, spinal arachnoid web versus cyst, vascular malformations, and beyond. The structure of the spinal cord itself was poorly visualized. The pial lining of the spinal cord caused a significant degree of signal reflection. Similarly, nerve roots created a shadow beyond their structure.

Moving forward, certainly a spinal-dedicated device is necessary. The optimal device would have a larger field-of-view and depth of tissue penetration. The catheter diameter should be a small as possible, ideally <1-mm in diameter and does not require microguidewire supported navigation. Recently, researchers developed a neurointerventional specific OCT device uniquely designed to address the difficulties of cerebrovascular imaging.^19^ We envision several applications for such a device. With respect to spinal cord injury, Badhiwala et al.^20^ recently showed that surgical decompression within 24 h of spinal cord injury resulted in improved functional outcome, with substantial heterogeneity among patients. Post-traumatic intrathecal structural OCT imaging may provide information regarding the tissue integrity and residual function. Mesquita et al.^21^ showed using a prototype optical fiber device that measurements of blood flow and oxygenation to the spinal cord can be recorded in real time under various pathologic conditions causing ischemia. Armed with the combination of structural and hemodynamic information in a spinal dedicated device, clinicians could determine if surgery would be advantageous and the timing of surgery in the setting of compression causing reversible ischemia.

Furthermore, in patients with spinal cord tumors, structural optical imaging could provide clear definitions of tissue boundaries therefore guiding extent of resection, which is challenging in spinal surgery. We also envision that intrathecal imaging could easily identify and differentiate arachnoid webs, ventral cord herniation, and arachnoid cysts. Clinicians currently have difficulty differentiating between these pathologies and often MRI combined with CT myelography is required, which are invasive and time-consuming diagnostic tests. In this preclinical study, arachnoids bands and dentate ligaments were well visualized, and therefore, structural OCT should be able to differentiate these pathologies. Similarly, for arachnoiditis, OCT could reveal regions of thickened tissue and bands therefore confirming the inflammatory diagnosis. Finally, large arteries and draining veins of dural arteriovenous fistulas or arteriovenous malformations could be identified with both structural and hemodynamic optical imaging. Localization and characterization of the lesions is often difficult and a minimally invasive modality could provide useful for both diagnoses, possibly stratify rupture risk, and assess for post-treatment cure.

The application of endovascular OCT in spinal imaging has several limitations. The repurposing of a device, from coronary to spinal imaging, will inherently come with restrictions. The first limitation is the field-of-view of the OCT device. Given that the diameters of coronary arteries are generally a few millimeters, the current device will not be able to fully image a human spinal cord with a diameter of 15 mm at the C5 level.^22^ This would lead to missing important anatomic details and pathology. Another limitation is the depth of penetration of the current device. An inherent limitation of the detection of single-scattered infrared light limits tissue depth penetration to ∼3  mm. This limited penetration will not capture the entire spinal cord. The OCT catheter used is not currently designed to image tissue such as the spinal cord or nerves but rather blood vessel luminal atherosclerosis, dissections, thrombus, and plaques.

## 5 Conclusion

Intrathecal spinal canal imaging using endovascular OCT was feasible in this preclinical animal study. The repurposing of an endovascular device for spinal imaging comes with limitations including decreased field-of-view and depth of tissue penetration. A spine-specific OCT device is necessary moving forward and could propel a new field of minimally invasive spinal imaging.
